# Google for Sexual Relationships: Mixed-Methods Study on Digital Flirting and Online Dating Among Adolescent Youth and Young Adults

**DOI:** 10.2196/10695

**Published:** 2019-05-16

**Authors:** James Lykens, Molly Pilloton, Cara Silva, Emma Schlamm, Kate Wilburn, Emma Pence

**Affiliations:** 1 Center for Research and Education on Gender and Sexuality San Francisco State University San Francisco, CA United States; 2 Youth Tech Health Oakland, CA United States

**Keywords:** youth, sexual health, online dating, adolescent health

## Abstract

**Background:**

According to a 2015 report from the Pew Research Center, nearly 24% of teens go online almost constantly and 92% of teens are accessing the internet daily; consequently, a large part of adolescent romantic exploration has moved online, where young people are turning to the Web for romantic relationship-building and sexual experience. This digital change in romantic behaviors among youth has implications for public health and sexual health programs, but little is known about the ways in which young people use online spaces for sexual exploration. An examination of youth sexual health and relationships online and the implications for adolescent health programs has yet to be fully explored.

**Objective:**

Although studies have documented increasing rates of sexually transmitted infections and HIV among young people, many programs continue to neglect online spaces as avenues for understanding sexual exploration. Little is known about the online sexual health practices of young people, including digital flirting and online dating. This study explores the current behaviors and opinions of youth throughout online sexual exploration, relationship-building, and online dating, further providing insights into youth behavior for intervention opportunities.

**Methods:**

From January through December 2016, an exploratory study titled TECHsex used a mixed-methods approach to document information-seeking behaviors and sexual health building behaviors of youth online in the United States. Data from a national quantitative survey of 1500 youth and 12 qualitative focus groups (66 youth) were triangulated to understand the experiences and desires of young people as they navigate their sexual relationships through social media, online chatting, and online dating.

**Results:**

Young people are using the internet to begin sexual relationships with others, including dating, online flirting, and hooking up. Despite the fact that dating sites have explicit rules against minor use, under 18 youth are using these products regardless in order to make friends and begin romantic relationships, albeit at a lower rate than their older peers (19.0% [64/336] vs 37.8% [440/1163], respectively). Nearly 70% of youth who have used online dating sites met up with someone in person (44.78% [30/67] under 18 vs 74.0% [324/438] over 18). Focus group respondents provided further context into online sexual exploration; many learned of sex through pornography, online dating profiles, or through flirting on social media. Social media played an important role in vetting potential partners and beginning romantic relationships. Youth also reported using online dating and flirting despite fears of violence or catfishing, in which online profiles are used to deceive others.

**Conclusions:**

Youth are turning to online spaces to build sexual relationships, particularly in areas where access to peers is limited. Although online dating site use is somewhat high, more youth turn to social media for online dating. Sexual relationship-building included online flirting and online dating websites and/or apps. These findings have implications for future sexual health programs interested in improving the sexual health outcomes of young people. Researchers may be neglecting to include social media as potential sources of youth hookup culture and dating. We implore researchers and organizations to consider the relationships young people have with technology in order to more strategically use these platforms to create successful and youth-centered programs to improve sexual health outcomes.

## Introduction

Recent increases in personal technology ownership among youth [1] combined with the developmental stage of sexual exploration and identity development [2] have changed the way young people prefer to build relationships and undergo sexual exploration. Websites and dating apps are popular spaces in which to develop sexual relationships and locate sexual health information [3-4], experiment with sexual play [2], discover pornography, and begin dating [5]. Previous studies have examined the role of online sexual exploration among adolescents as a time of identity construction and expression [6-7]; a decade ago, early chat rooms and social media platforms provided ideal ways for young people to connect and construct identities. However, due to constantly changing online tools for social connection and dating, few studies have recently explored the new ways in which adolescents use and describe their online use for sexual exploration and relationship-building. In addition, few studies have focused specifically on the use of online dating websites among youth younger than 18 years; instead, most studies have either focused on the victimization of minors online and the moral panics around youth sexuality and new media use or have relied on a monolithic description of youth that fails to consider developmental differences among ages in regard to sexual health research [8].

An exploration into the use of online dating and social media for sexual exploration among youth is timely considering the increased rates of sexually transmitted infections and HIV among youth today [9]. As these trends continue to increase, it is vital for researchers and program developers to consider the unique interactions that young people have with sexual health and relationships online in order to create successful programs, particularly due to the rapidly changing landscape of online dating and social media platforms. Further, a historical failure to focus on minor youth (younger than 18 years) differences and relying on traditional approaches to understanding sexual and romantic behaviors of young people may be insufficient to fully capture the sexual health implications of online sexual exploration and relationship development.

While many projects have found success bringing sexual health programs online [10-12], and recent studies have begun to incorporate a more complicated definition of youth [13], an examination of the unique relationships young people have with their sexual exploration and relationships online has yet to be fully explored. In particular, few studies have focused on the amalgamation of online dating and social media [14] or the level of trust and engagement that youth have invested in online dating and flirting, particularly in the context of public health. Exploratory studies are needed to remain up to date on youth trends and behaviors in order to locate opportunities for health interventions and future research.

In response, the TECHsex national research project examined the online sexual exploration habits of young people in order to paint a broader picture of online sexual relationships, including online dating, trusted websites, age differences, behaviors, and online flirting. The implications of these data demonstrate that future programs should consider the varied experiences, levels of trust, age, and behaviors of youth prior to developing sexual health programs if they wish to yield greater health outcomes among young people.

## Methods

In this exploratory study, we used a mixed-methods approach to capture a broad understanding of sexual exploration and relationships online among young people. The study received human protections approval from the Quorum Institutional Review Board.

### Quantitative Phase

A national self-report survey, hosted on the online survey software Qualtrics, was conducted from September 2015 through July 2016. A total of 1500 youth ages 13 to 24 years (average age 19.70 years) responded; 22.40% (336/1500) of respondents were younger than 18 years.

Survey questions were determined by a comprehensive literature review of recent reports and articles on youth technology use, positive youth development [15], sexual exploration, and sexual health in the United States. The survey asked detailed demographic and behavioral questions on technology use, sexual behavior online, romantic relationship behavior online, and trust in dating and flirting online. Data reported here are a subset of our complete TECHsex report [16], which includes additional data on other youth technology trends. Data detailed here are self-reported responses to closed-ended survey questions. We analyzed these data using descriptive statistics including cross-tabulations and frequencies.

### Qualitative Phase

In addition to the online quantitative survey, semistructured qualitative focus groups were conducted to contextualize quantitative findings. Findings from the quantitative survey directly influenced the creation of the semistructured interview guide for focus groups. Focus groups were conducted in the south, west, midwest, and northeast United States in the following cities: Berkeley, CA; Oakland, CA; Tunica, MS; Birmingham, AL; Newark, NJ; New Orleans, LA; and Chicago, IL. Study sites were chosen for regional diversity and documented sexual health needs [17-18]. Focus groups lasted approximately 90 minutes. All participants were required to sign a consent form (if minors, participants were required to have consent forms signed by parents or guardians). Efforts were made to increase the diversity of respondents by working with local youth-serving organizations to recruit at least half of the respondents as youth of color and/or lesbian, gay, bisexual, transgender, and queer youth. Two focus groups per site were conducted and stratified by age, one for participants younger than 18 years and the other for those over 18 years.

Focus groups were audio recorded, transcribed, and de-identified for confidentiality purposes. Using a thematic analysis approach [19], the lead authors identified major themes across focus groups, including sexual health information online, online dating, and digital flirting. Using the Cohen kappa statistic, interrater reliability was established between two coders with an overall agreement percentage of 97.45% (range 73.90% to 100% agreement across all codes) [20]. Quotes from focus groups are included to contextualize quantitative data from the national survey.

### Recruitment

Participants for the national survey were recruited via community partners with flyers and postcards, as well as through panel companies (organizations that enroll interested users based on reported demographics and provide small incentives for survey completion). Participants received US $5 in digital currency for survey completion. Focus group participants were recruited through community partners at each site using traditional methods of recruitment including flyers, postcards, and social media posts in youth-centric locations including school campuses, clinics, and public areas. Focus group participants were compensated with a US $25 Visa gift card for their participation.

### Quantitative National Survey

A sample of 1500 youth across the United States, ages 13 to 24 years (average age 19.7 years), completed the online survey. All four regions as defined by the US Census Bureau [21] were represented: west (414/1500, 27.60%), midwest (362/1500, 24.13%), south (410/1500, 27.33%), and northeast (314/1500, 20.93%). A total of 22.40% (336/1500) of respondents were younger than age 18 years. The race and ethnicity breakdown of the sample was 57.60% (864/1500) white, 20.13% (302/1500) black/African American, 5.33% (70/1500) American Indian or Alaska Native, 9.33% (140/1500) Asian, 1.46% (22/1500) Native Hawaiian or other Pacific Islander, and 11.40% (171/1500) another race not listed. Nearly 24% (356/1500, 23.73%) of the sample identified as Hispanic or Latino. The gender breakdown was 62.00% (930/1500) women, 33.67% (505/1500) men, and 4.30% (65/1500) transgender-spectrum (consisting of individuals who identify as a different gender than their sex assigned at birth including transgender woman, transgender man, and genderqueer). Further information on employment status and education are reported in Table 1.

### Qualitative Focus Groups

A total of 66 youth participated in the focus groups. The demographic breakdown by race was 81% (53/66) black/African American, 3% (2/66) Asian, 2% (1/66) American Indian or Alaska Native, 2% (1/66) Native Hawaiian or other Pacific Islander, 5% (3/66) white, and 2% (1/66) another race not listed. Approximately 20% (13/66) of the sample identified as Hispanic or Latino. The gender breakdown was 52% (34/66) women, 42% (28/66) men, and 6% (4/66) transgender-spectrum. Further information on employment status and education are reported in Table 1.

**Table 1:** Sample demographics.

Characteristic		Quantitative survey (n=1500), n (%)	Qualitative focus group (n=66), n (%)
**Gender**			
	Male	495 (33.0)	27 (40.9)
	Female	930 (62.0)	34 (51.5)
	Transgender-spectrum	75 (5.0)	5 (7.6)
**Age**			
	Minor (younger than 18 years)	336 (22.4)	28 (42.4)
	Adult (18 years and older)	1164 (77.6)	38 (57.6)
**Highest level of education**			
	Junior high or middle school	72 (4.8)	7 (10.6)
	Some high school	223 (14.8)	24 (36.4)
	High school diploma	444 (29.6)	14 (21.2)
	Technical school	39 (2.6)	0 (0)
	Some college/university	472 (31.5)	11 (16.7)
	Undergraduate degree	148 (9.9)	4 (6.1)
	Graduate degree	102 (6.8)	6 (9.1)
**Employment**			
	Unemployed	247 (16.4)	25 (38.9)
	Full-time student	585 (39.0)	20 (30.3)
	Part-time student (less than 30 hours)	79 (5.2)	7 (10.6)
	Part-time student (more than 30 hours)	84 (5.6)	4 (6.1)
	Part-time	107 (7.1)	4 (6.1)
	Full-time	256 (17.1)	3 (4.5)
	Contract, freelance, or temporary employee	8 (0.5)	0 (0)
	Self-employed	26 (1.7)	0 (0)
	Homemaker (at home without children)	28 (1.8)	0 (0)
	Stay-at-home parent (at home with children)	42 (2.8)	1 (1.5)
	Other	38 (2.5)	2 (3.0)

## Results

### Using Online Dating and Online Flirting for Sexual Exploration

Current perceptions of sexual exploration and online romantic relationship formation are complicated among youth. While online romantic relationships and dating were certainly present, youth across all ages held a pervasive fear of dating violence and cyber abuse; despite this fear, many used online dating sites as a place to find and begin romantic relationships. Participants often were forced to reconcile these warring feelings in order to continue exploring sexual relationships online and accessing online dating spaces. For many younger adolescents (younger than age 18 years) using social media as an alternative space for online dating allows them to bypass feelings of fear due to the heightened level of trust in these platforms. The following results focus on these two themes, including benefits and fear of technology for sexual exploration and relationships. Both themes are addressed below, supported by triangulated data from the national survey and regional focus groups.

### Benefits of Technology for Sexual Exploration and Relationships

#### Quantitative Survey

**Figure 1:**
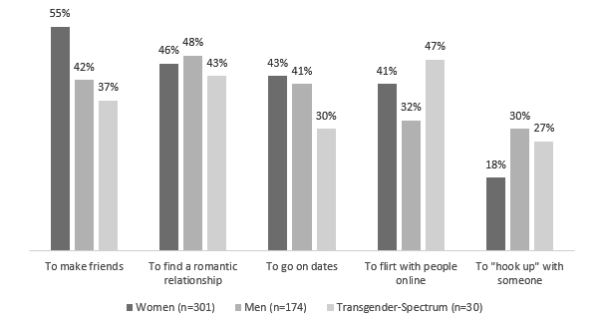
Reasons for using a dating app by proportion and gender.

Youth are forming romantic relationships online and using dating apps frequently. About 34.00% (510/1500) of survey respondents reported that they use dating sites/apps or have in the past. Despite the fact that these dating sites have explicit rules against minor use, under 18 youth are using these products in order to make friends and begin romantic relationships, albeit at a lower rate than their older peers (19.0% [64/336] vs 37.80% [440/1163], respectively). Nearly 80.0% (404/505) of youth who have used online dating sites met up with someone in person (45% [30/67] under 18 vs 74.0% [324/438] over 18). The most popular dating platforms were Tinder, OkCupid, and Match, with Tinder most popular among both minors (27/67, 40%) and over 18 youth (227/438, 51.8%). Of those who reported current or past use of dating apps, under 18 youth reported that they primarily used them to make friends online (41/67, 61%) compared with over 18 youth primarily using them for finding romantic relationships (214/438, 48.9%) (see Figure 1 for further breakdown by gender). About a third (58/175, 33.1%) of men who use or have used dating sites reported using them to hook up (ie, to first meet online and then decide on a location for a casual sexual encounter) with someone. In contrast, only 17.9% (54/302) of women and 27% (8/30) of transgender-spectrum youth reported using dating sites to hook up with someone. This relatively popular use of online dating has implications for health interventions; some current sexual health education programs that use dating apps to recruit and disseminate information have shown initial promise, particularly with sexual and gender minority youth [16].

Although the majority of youth do not use online dating websites (990/1500, 66.00%), youth are still building romantic relationships online by flirting with others through social media. Digital flirting often takes the form of comments, private messaging, innuendo emojis (ie, the eggplant or water squirt emoji to mimic a penis or ejaculation), or liking someone’s photos on social media. Nearly 41.00% (615/1500) of respondents said that they used social media to flirt with others. When asked how they flirted with others online, women were most likely to send messages to flirt with someone (280/346, 80.9%), men were most likely to like someone’s photos (191/239, 79.9%), and transgender-spectrum youth were most likely to message someone (33/39, 85%).

#### Qualitative Focus Groups

Within focus groups, online flirting was cited as the entry point for hookups and dating. Instagram, Facebook, and Snapchat were cited as places where youth could find others who have similar interests and begin conversations with potential partners. Youth explained that they flirt online to start a conversation with someone they are interested in. Usually youth flirt online with their extended network and can discover other people of interest by looking through the friend lists of people they already know, which could potentially lead to a sexual encounter. Because digital flirting often takes place over social media and behind a screen, it also allows youth to more confidently approach someone. As one over 18 youth in Oakland, CA, reports, online dating is a way to find romantic partners anywhere.

It’s pretty easy to find people that don’t live near you. So if you wanna find somebody without online dating you have to like go and actually find them. With online dating you can like search everywhere.

Oakland, CA, participant; over 18 years

Although the majority of focus group participants had experience with digital flirting or had flirted with others online, online dating sites (eg, Tinder or Grindr) elicited a more mixed response, particularly among youth under 18. Online dating sites were perceived as doors to hookup culture or a place to trick or “catfish” someone (ie, when someone has a false online dating profile with the intention of deceiving others). In rare cases, some youth did believe that you could develop romantic and long-term relationships online. However, reactions to online dating were typically negative and couched within general mistrust of using technology to meet someone.

I know like I have friends who do that, they just meet up with dudes [from Tinder] and like get them to buy bottles of stuff in exchange for like sex. And they will like sleep at their house and stuff like that for nights at a time and it’s just crazy.

Oakland, CA, participant; under 18 years

Another under 18 participant in Tunica, MS, shared fears of catfishing when using online dating.

Online dating, it’s very easy for you to lie. And they can just find where you live. Just show up where you’re at.

Tunica, MS, participant; under 18 years

Despite the overall negative reaction to online dating, participants did share some positive experiences with online dating. Some focus group participants were adamant about the positives of online dating, citing it as another place to potentially find the one. Older focus group participants were very aware of some of the dangers but also understood that online dating works for some people and can result in happy relationships. Participants who identified within the lesbian, gay, bisexual, and transgender-spectrum (LGBT) community were more likely to think highly of online dating as a place to find like-minded peers.

Most participants who decried online dating for fear of false information or threats to personal security reported similar online dating behaviors through social media flirting, where they often located potential partners and began romantic and sexual relationships with those they met online. For these participants, the vetted experience of social media through connected peers eased the fear of potential violence, catfishing, or abuse.

## Discussion

### Principal Findings

Results indicate that online spaces often serve as primary avenues to begin romantic relationships and foster sexual identities through online flirting and dating, regardless of minor or adult status. By building these relationships online, young people are fully involved in the many facets of online sexual exploration including developing first sexual relationships and accessing online hookup culture. For many, this is a positive experience, particularly for LGBT respondents and some rurally located participants who would otherwise not be able to connect with peers. For others, online dating was interpreted as dangerous and to be avoided, despite reporting the same online activities through social media to begin romantic relationships. Due to this fear, many young people have migrated to social media as a safer online dating option, possibly due to the increased familiarity and visibility of such platforms. This is particularly true for under 18 focus group participants, who were far more likely to consider dating those they met through social media than online dating.

### Limitations

The nature of our nonrepresentative sample inhibits us from being able to generalize our findings to a larger youth population. However, the large sample size provides initial steps in understanding the way in which young people use and trust the internet and social media for romantic relationship-building and sexual exploration. In addition, because the quantitative survey was hosted online, it may be that some youth without access to personal internet-enabled devices were left out of the study. Future studies should consider representative samples, particularly among gender identity and age, as well as how to include young people who may not be reached due to location or access, including rural youth. In addition, due to the exploratory nature of this study, this paper does not provide data on predictive behavior of sexual exploration and relationship-building online among youth.

### Conclusion

Results from this study indicate that online dating platforms and social media are both promising spaces for health interventions and information dissemination. Researchers and organizations should consider the unique relationships between young people, sexual health and relationships, and trusted online spaces to create tailored and effective online health programs. These complicated relationships illustrate that the internet and social media are ideal platforms for program implementation, but researchers must be conscious of the varying amounts of trust that youth place in these spaces. In particular, these data highlight the need for dating-based interventions even among minor youth, who may be left out of such programs due to the misconception that they are not using online dating for sexual exploration and relationship formation because of legal restraints. In addition, online dating is a useful platform for interventions, but researchers must not exclude dating and hookup practices on social media, which are widely used and more trusted among youth.

In addition, the interest and participation in online dating and digital flirting may provide a window into the sexual health and behaviors of young people online and could provide opportunities for enhancing healthy relationship formation. One 2017 study explored the nature of healthy online dating dynamics, which could be an important new field of sexual health education for young people [22]. Other studies have highlighted the intersection of online dating and cyberbullying [23], further stressing the need for contemporary relationship education in order to enhance sexual and mental wellness among youth.

In sum, young people are clearly using online spaces for building romantic relationships, even when they reject traditional forms of online dating. It should be further noted that this data does not provide a condemnation of these practices, but simply demonstrates the complicated relationship that youth have with online sexual exploration and relationship development. In fact, these online practices can provide many benefits as well as risks, and are vital considerations for future health programs. These findings could have implications for sexual health and risk for future programs aimed at enhancing youth sexual health. A nuanced approach that considers age difference among youth and the amount of trust in the dating platform will provide a more tailored approach to reducing negative health outcomes.

## Abbreviations

LGBT: lesbian, gay, bisexual, and transgender-spectrum
