# Correction: Google for Sexual Relationships: Mixed-Methods Study on Digital Flirting and Online Dating Among Adolescent Youth and Young Adults

**DOI:** 10.2196/14815

**Published:** 2019-06-21

**Authors:** James Lykens, Molly Pilloton, Cara Silva, Emma Schlamm, Kate Wilburn, Emma Pence

**Affiliations:** 1 Center for Research and Education on Gender and Sexuality San Francisco State University San Francisco, CA United States; 2 Youth Tech Health Oakland, CA United States

The manuscript “Google for Sexual Relationships: Mixed-Methods Study on Digital Flirting and Online Dating Among Adolescent Youth and Young Adults” (JMIR Public Health Surveill 2019;5(2):e10695) was initially published with two separate headings for the Methods section. This error has now been corrected and the second heading has been removed.

The correction will appear in the online version of the paper on the JMIR website on June 21, 2019, together with the publication of this correction notice. Because this was made after submission to PubMed, PubMed Central, and other full-text repositories, the corrected article also has been resubmitted to those repositories.

